# The asset-vulnerability trap: climate shocks and health-seeking behavior for chronic diseases in Somalia

**DOI:** 10.3389/fpubh.2026.1869640

**Published:** 2026-07-09

**Authors:** Abdirashid Jama Abdullahi, Abdirizak Mohamed Moumin, Khadar Mowlid Abdi

**Affiliations:** Faculty of Business and Economics, Amoud University, Borama, Somaliland, Somalia

**Keywords:** chronic diseases, climate vulnerability, Heckman probit model, livestock loss, non-communicable diseases, pastoralist health

## Abstract

Somalia faces a dual crisis of high chronic disease burden and extreme climate vulnerability. This study examines the predictors of health-seeking behavior (HSB) for chronic diseases among Somali households. Using data from the 2020 Somali Demographic and Health Survey (SDHS), survey-weighted probit model was applied to a sample of 69,998 individuals to analyze predictors of regular treatment-seeking behavior. Approximately 6.6% of the population reported a chronic disease, with 48.8% of those individuals seeking regular treatment. Wealth (AME = 0.094, *p* < 0.001) and urban residence (AME = 0.078, *p* < 0.001) were the strongest predictors of HSB. Secondary education increased HSB by 6.2 percentage points (*p* = 0.012). Conversely, climate-induced livestock loss significantly hindered HSB, reducing the probability of treatment-seeking by 3.4 percentage points (*p* = 0.035). Internet access was not a significant predictor. Climate shocks create an economic barrier that prevents vulnerable households from managing chronic conditions. Policy interventions should focus on integrating NCD care into climate adaptation strategies and reducing the rural–urban health gap.

## Introduction

Health-seeking behavior (HSB) is defined as the sequence of remedial actions individuals undertake to rectify perceived ill-health, a process deeply embedded within the Social Determinants of Health (SDH) framework. Achieving universal health coverage and ensuring healthy lives [Sustainable Development Goal (SDG) 3] requires a strategic alignment with climate action (SDG 13), as environmental stability is a prerequisite for functional health systems. The SDH framework emphasizes that health outcomes are shaped by the broader economic, social, and political conditions in which people live and age (1). In fragile and conflict-affected settings, these determinants are further complicated by the “silent crisis” of non-communicable diseases (NCDs), where the lack of continuous care in unstable environments leads to high rates of preventable complications (2). In the context of chronic disease management, HSB becomes a critical determinant of long-term survival; however, for populations in climate-vulnerable regions, this behavior is frequently disrupted by environmental stressors that undermine both the physical accessibility and the financial viability of healthcare.

The Vulnerability and Adaptation (V&A) framework provides a foundational analytical tool to understand these disruptions, conceptualizing vulnerability as a function of exposure, sensitivity, and adaptive capacity (1) Globally, the burden of disease is increasingly sensitive to climatic shifts, with environmental factors accounting for as much as 94% of diarrheal and waterborne disease cases in low-income settings (3). In fragile states, the resilience of the health system is often too low to absorb the shock of climate-induced disease surges, leading to a total collapse of NCD management protocols (4). Recent studies in Nepal and Bangladesh have demonstrated that even minor increases in mean temperature and erratic rainfall patterns result in a considerable rise in disease incidence, which in turn strains the adaptive capacity of households (3, 5, 6).

In the Horn of Africa, Somalia represents one of the most complex intersections of climate vulnerability and public health fragility. The country is characterized by a high dependence on pastoralism, making its population uniquely sensitive to climate-induced hazards. According to the Somali Demographic and Health Survey (SDHS) 2020, environmental shocks often lead to the massive loss of livestock, which serves as the primary capital for Somali households. This loss of pastoral viability is a significant “push factor” that forces households into a state of involuntary adaptation (7).

In the Somali context, livestock loss serves as the most accurate proxy for climate-induced shocks, representing the “mobile bank” of the household. While climate change manifests through various environmental shifts, livestock depletion uniquely captures the household-level economic devastation caused by droughts and floods in pastoralist economies. It serves as a more direct determinant of financial capacity for healthcare than aggregate regional rainfall or temperature data. For these populations, livestock is the primary capital required to fund medical expenses (8). As households lose their primary assets to drought or floods, they face a “dual burden”: the compounding effects of inadequate healthcare infrastructure and the adverse impacts of climate change, which often force a choice between immediate survival and long-term medical treatment (9).

The intersection of education and gender roles further complicates health-seeking trajectories for chronic conditions. Evidence from coastal Ghana suggests that the patriarchal nature of decision-making can significantly impact the management of diseases, as men often control the economic resources necessary for healthcare access (3). In Somalia, specific barriers to NCD care—such as diabetes and hypertension—are exacerbated by a lack of specialized providers and the high cost of long-term medication (10). Simultaneously, education serves as a critical proxy for health literacy. Individuals with higher formal education are generally better equipped to understand the necessity of regular treatment for chronic conditions (3). In fragile contexts, health literacy is often a stronger predictor of HSB than geographic proximity, as educated individuals are more likely to navigate the fragmented Somali health landscape to find professional care (11).

Economic barriers, specifically trust, affordability, and availability, remain the primary factors influencing the choice between modern pharmaceutical drugs and traditional remedies. In many climate-vulnerable communities, “over-the-counter” (OTC) drugs and home-made traditional remedies are the most common first management actions due to the perceived high cost of formal health facilities (1, 3). The financial catastrophe associated with chronic disease treatment in low-income settings often leads to “distress selling” of remaining assets, further deepening household poverty (12, 13). When climate shocks occur—specifically the loss of livestock—household wealth is further depleted, creating a “vulnerability trap.” This depletion of standardized wealth scores significantly reduces the probability of seeking regular medical treatment, as households are forced to save limited resources for food rather than healthcare (3).

Despite the wealth of data provided by the SDHS 2020, there remains a significant gap in understanding the specific predictors of HSB for chronic diseases in Somalia’s climate-vulnerable households. Most existing literature focuses on macro-level impacts like food security or acute infectious outbreaks, with less attention paid to how individual socio-demographic factors interact with specific environmental shocks to determine long-term health trajectories (1) This study addresses this gap by utilizing a Heckman probit model to analyze the predictors of regular treatment-seeking behavior while accounting for selection bias. By examining the impact of education, standardized wealth, and climate-induced asset loss, this research aims to provide actionable insights for developing climate-resilient health policies in Somalia.

## Methodology

### Data sources

The analysis is based on secondary data from the 2020 Somalia Demographic and Health Survey (SDHS), a nationally representative survey implemented by the Directorate of National Statistics of the Federal Government of Somalia. The SDHS provides robust data on a wide range of demographic, socioeconomic, and health indicators. The choice of 2020 data is justified as it represents the most recent and comprehensive nationally representative dataset available for Somalia, capturing critical health-seeking metrics alongside environmental shock indicators. Study population and regional structure.

The study population encompasses individuals residing in households across all 16 administrative regions of Somalia, covering urban, rural, and nomadic strata. This broad inclusion is vital for capturing the diverse landscape of climate vulnerability and health infrastructure in a fragile state setting.

### Sample and weighting

The initial survey included all usual residents. The analytical sample was restricted to individuals aged 6–95 with complete data across all predictor variables, resulting in a final sample of 69,998 individuals (Table 1). While chronic diseases predominantly manifest in adults, individuals aged 6 and above were retained in the denominator because early-onset conditions (e.g., Type 1 diabetes, rheumatic heart disease) necessitate regular health-seeking behaviors that are entirely mediated by the household’s socioeconomic status and asset vulnerability. Individuals under 6 were excluded as their healthcare utilization is typically captured under separate maternal and child health indicators. To ensure national representativeness and account for household-level clustering of variables (e.g., wealth, livestock loss, clean fuel), all analyses were adjusted using the SDHS multi-stage stratified cluster sampling design. The primary sampling units (PSUs) were the enumeration areas (HV001), and stratification was based on geographic regions (HV024). Individual survey weights (HV005) were applied to compute design-adjusted standard errors, mitigating the risk of overstating statistical significance for household-level predictors.

**Table 1:** Descriptive statistics of the study population.

Variable	*M*	SD	Min	Max
Chronic disease (selection)	0.066	0.226	0	1
Regular treatment (outcome)	0.488	0.500	0	1
Internet access	0.088	0.276	0	1
Secondary education or higher	0.095	0.293	0	1
Clean cooking fuel	0.616	0.485	0	1
Climate shock (livestock loss)	0.357	0.479	0	1
Urban residence	0.454	0.498	0	1
Age	19.30	18.24	0	95
Wealth score (standardized)	0.000	1.00	−2.00	3.13

*N* = 69,998. Wealth score was standardized to a mean of 0 and a standard deviation of 1. Variables coded as 0 or 1 represent proportions (e.g., 0.066 = 6.6%).

### Study variables

#### Selection and outcome variables

The primary outcome of interest is Regular Treatment-Seeking Behavior, operationalized as a binary variable coded as “1” if an individual with a chronic condition reported seeking regular medical treatment or advice from a professional health provider, and “0” otherwise.

However, health-seeking behavior can only be observed among individuals who perceive themselves to have a health problem. Therefore, a Selection Variable was defined as the presence of a self-reported Chronic Disease (e.g., diabetes, hypertension, or kidney disease). This dual-variable approach allows for the correction of selection bias, as individuals who seek treatment are not a random subset of the population but are selected based on their disease status.

#### Predictor variables

Predictor variables were selected based on the Social Determinants of Health (SDH) and the Vulnerability and Adaptation (V&A) frameworks.

- Socio-demographic Factors: Age, secondary education or higher (as a proxy for health literacy), and urban vs. rural residence.
- Economic Factors: Standardized wealth score (standardized to a mean of 0 and SD of 1) and access to internet (proxy for digital health information access).
- Environmental/Climate Vulnerability: The primary climate stressor was operationalized as Climate Shock (Livestock Loss), a binary indicator reflecting the loss of primary household capital due to drought or floods.
- Infrastructure Proxy: Use of clean cooking fuel was included as an indicator of household environmental quality and socioeconomic stability.

### Statistical analysis

The statistical analysis followed a three-stage empirical strategy. First, descriptive statistics were calculated to characterize the study population (Table 1). Second, bivariate associations were examined using Pearson’s chi-square tests to identify significant correlates of chronic disease and treatment-seeking. To evaluate the predictors of regular treatment, a survey-weighted probit regression was utilized as the primary analytical model among the sub-population reporting a chronic disease (*N* = 4,617). Given the cross-sectional nature of the data, this model identifies robust associations rather than strict causal pathways. To test for potential selection bias—where treatment-seeking is only observable for those suffering from a chronic illness—a Heckman probit model was conducted as a sensitivity analysis. All analyses were conducted using Stata version 16.0.

## Results

As shown in Table 1, approximately 5.4% of the population reported suffering from a chronic disease. Among those with a chronic condition, 48.5% reported receiving regular treatment. Regarding the primary predictors, 9.5% of the sample had attained secondary education or higher, while only 8.3% had access to the internet. Notably, 35.7% of households reported a significant climate shock in the form of livestock loss due to drought or floods.

In Table 2, initial bivariate analyses revealed stark disparities in health-seeking behavior, with secondary education significantly increasing the likelihood of receiving regular treatment (65.0% vs. 52.4%, *p* < 0.001) and climate-induced livestock loss significantly reducing it (44.3% vs. 56.7%, *p* < 0.001). To account for confounding variables and isolate the true drivers of care, a standard probit regression was employed. This multivariate model confirmed that household wealth and urban residence are the strongest positive determinants of regular treatment (*p* < 0.001$), while secondary education remains a significant facilitator (*p* = 0.045). Crucially, experiencing a climate shock retains a significant negative effect on health-seeking behavior (coefficient = −0.106, *p* = 0.018$), mathematically demonstrating that environmental asset vulnerability directly impedes healthcare access even when controlling for baseline wealth and education. Conversely, internet access did not emerge as a statistically significant predictor ($*p* = 0.150$), suggesting digital access alone does not bridge the care gap in this context. Finally, to ensure these findings were not artificially skewed by selection bias—given that treatment-seeking is contingent upon first having a chronic disease—a Heckman probit sensitivity analysis was conducted. While the selection equation confirmed that socioeconomic factors dictate who reports an illness, the likelihood ratio test of independent equations was not statistically significant (*p* = 0.480). This lack of selection bias validates the standard probit estimates as the robust, primary framework for interpreting these healthcare dynamics.

**Table 2:** Standard probit estimates.

Variable	Coefficient	SE	*z*	*p*
Outcome: regular treatment				
Internet access	0.105	0.073	1.44	0.150
Secondary education or higher	0.137**	0.068	2.00	0.045
Climate shock	−0.106**	0.045	−2.37	0.018
Urban residence	0.190***	0.047	4.08	<0.001
Wealth score (standardized)	0.260***	0.027	9.78	<0.001
Constant	−0.116***	0.039	−2.97	0.003

*N* = 4,617. The sample is restricted to individuals reporting a chronic disease. SE, Standard error *p < 0.10, **p < 0.05, ***p < 0.01.

In Table 3, to provide a more intuitive interpretation of the results, Average Marginal Effects (AME) were calculated to determine the discrete change in the probability of seeking regular treatment (Table 3). The results indicate that socioeconomic factors are the strongest predictors of health-seeking behavior. A one-standard-deviation increase in the wealth score was associated with a 9.4 percentage point increase in the probability of seeking regular treatment (AME = 0.094, *p* < 0.001). Education also played a critical role; individuals with at least a secondary education were 6.2 percentage points more likely to seek treatment (AME = 0.062, *p* = 0.012). Conversely, climate vulnerability acted as a significant barrier. Households that experienced livestock loss due to drought or floods were 3.4 percentage points less likely to seek regular treatment (AME = −0.034, *p* = 0.035). Urban residence significantly increased the probability of treatment by 7.8 percentage points (AME = 0.078, *p* < 0.001). Interestingly, while internet access was positively associated with treatment seeking, the effect was not statistically significant (AME = 0.032, *p* = 0.228).

**Table 3:** Average marginal effects.

Variable	dy/dx (AME)	SE	*z*	*p*
Internet access	0.032	0.026	1.21	0.228
Secondary education or higher	0.062**	0.024	2.51	0.012
Climate shock	−0.034**	0.016	−2.11	0.035
Urban residence	0.078***	0.016	4.66	<0.001
Age	0.002***	0.000	6.36	<0.001
Wealth score (standardized)	0.094***	0.011	8.00	<0.001

AME, Average marginal effect; SE, Standard error. AME represents the discrete change in the probability of seeking regular treatment.

## Discussion

The findings of this study reveal a significant “vulnerability trap” within Somali households, where climate-induced capital loss is strongly associated with reduced health-seeking behavior (HSB) for chronic conditions. Our results indicate that climate shocks, specifically livestock loss, reduce the probability of seeking regular treatment by 3.4 percentage points (*p* = 0.035). This is further evidenced by the fact that a one-standard-deviation increase in wealth was associated with the largest increase in HSB (9.4 percentage points). This aligns with the “Asset-Vulnerability Nexus” described by Islam et al. (1), suggesting that when climate shocks deplete a household’s “mobile bank”—livestock—medical expenses for non-communicable diseases (NCDs) are the first to be sacrificed in favor of immediate survival needs. For Somali pastoralists, the loss of livestock is not merely an economic blow but a total erosion of the social safety net that facilitates health access (14). This is further evidenced by the fact that a one-standard-deviation increase in wealth was associated with the largest increase in HSB (8.8 percentage points), confirming that in fragile settings, regular healthcare for chronic conditions remains a luxury accessible primarily to those with stable economic buffers (13).

Furthermore, the strong positive impact of secondary education (6.2 percentage points) and urban residence (7.8 percentage points) underscores the profound socioeconomic and geographic divide in the Somali health landscape. Education acts as a critical proxy for health literacy, empowering individuals to navigate the fragmented and often unregulated Somali health system to find professional care (3, 9), this Urban Advantage reflects the highly privatized nature of the Somali health system, where specialized NCD care is heavily centralized in cities like Mogadishu and Hargeisa, while rural and nomadic populations remain systematically underserved (1). This geographic disparity is particularly dangerous for NCD management, which requires continuous, long-term interaction with health providers—a requirement that is nearly impossible to meet for rural households facing frequent climatic disruptions and geographic isolation (15) Interestingly, while digital information access is often touted as a solution for health gaps in developing nations, internet access was not a statistically significant predictor of HSB in this study (*p* = 0.310). This suggests that digital health interventions have not yet reached a critical mass in Somalia, likely due to the low overall penetration rate (8.3%) and the “digital divide” that favors urban elites. In fragile states, traditional health outreach and community-based education remain more viable than digital platforms for improving HSB (11). The non-significance of the Heckman Rho (*p* = 0.441) further suggests that while selection bias was not a major statistical driver in this specific sample, the socioeconomic barriers to even being diagnosed with a chronic disease (the selection equation) remain high, particularly for those with low education and wealth (2). Future research should extend this framework to include cross-regional comparative studies in other climate-vulnerable nations. Such comparative analyses would enhance the generalizability of these findings and deepen the global understanding of how climate-induced asset vulnerability influences health behaviors in fragile states.

### Policy implications

The findings of this study provide critical evidence for the integration of climate adaptation and health system strengthening in Somalia. Based on the empirical results, the following policy interventions are recommended:

#### Climate-linked financial protection for health

The significant negative impact of livestock loss on health-seeking behavior (AME = −0.030) suggests that climate shocks act as a direct economic barrier to chronic disease management. To protect the “mobile bank” of pastoralist and rural households, the Federal Government of Somalia and international partners should develop weather-indexed health insurance or emergency health vouchers. These financial instruments should be automatically triggered during documented periods of drought or flash floods, providing households with the liquidity needed to maintain regular medical treatment for NCDs without resorting to the “distress selling” of remaining assets.

#### Decentralization of NCD care to rural and nomadic strata

The pronounced “Urban Advantage” (AME = 0.081) indicates a systemic neglect of rural and nomadic populations in the Somali health landscape. Policy efforts must move beyond the centralization of specialized care in Mogadishu and Hargeisa. We recommend the integration of NCD management into the Primary Health Care (PHC) framework, specifically training Community Health Workers (CHWs) to monitor hypertension and diabetes in remote areas. Furthermore, the establishment of mobile NCD clinics that follow nomadic migratory routes is essential to ensure that climate-vulnerable populations are not forced to choose between their livelihoods and their health.

#### Strengthening health literacy through non-digital channels

While education was a strong facilitator of health-seeking behavior (AME = 0.061), Internet access was non-significant (*p* = 0.310), likely due to a digital divide where information is not yet widely available in the Somali language or tailored to nomadic health needs. This suggests that “Digital Health” is not yet a viable primary strategy for the most vulnerable Somali households. Policy interventions should instead focus on traditional health communication channels, such as radio broadcasts, community town halls, and engagement with religious and clan leaders. These campaigns should specifically target health literacy regarding the “silent crisis” of chronic diseases, emphasizing the importance of regular treatment even during periods of environmental stress.

#### Integration of health into national climate adaptation plans

Somalia’s National Adaptation Program of Action (NAPA) must be updated to include Chronic Disease Resilience. Currently, most climate-health policies focus on acute infectious outbreaks (e.g., cholera). However, our results show that the long-term management of chronic conditions is equally sensitive to climate shocks. By integrating NCD drug supply chains into disaster risk management protocols, the government can ensure that essential medications—such as insulin and anti-hypertensives—remain available and affordable even when climate-induced livestock loss occurs.

## Limitations

This study has some limitations. First, the data is cross-sectional, which allows for the identification of associations but precludes the establishment of strict causality. Beyond cross-sectional limitations, this study is subject to Recall Bias due to self-reporting. Furthermore, there is an “Unmet Need” bias: many low-wealth individuals may have a chronic disease but remain undiagnosed because they have never accessed a professional health provider, potentially underestimating the selection equation. Consequently, because this bias disproportionately excludes the most vulnerable individuals from the observable sample, the Average Marginal Effects (AMEs) reported for wealth and education likely underestimate the true magnitude of these socioeconomic disparities. Second, the presence of chronic diseases was self-reported by respondents rather than clinically verified, which may lead to recall bias or under-reporting. Finally, while the Heckman model addresses selection bias, there may be unobserved variables not captured by the SDHS 2020 that influence health-seeking behavior.

## Conclusion

This study concludes that health-seeking behavior for chronic diseases in Somalia is deeply correlated with climate stability and household wealth. The results suggest that climate-induced livestock loss is associated with a significant reduction in care-seeking, acting as a profound barrier to NCD management. To achieve Sustainable Development Goal 3 in the context of Somalia’s fragile state, it is imperative to decentralize NCD care into rural and nomadic strata and develop climate-linked social protection schemes, such as health vouchers for households suffering from documented livestock loss. Future policies must move beyond acute emergency response to build a climate-resilient health system that protects the most economically vulnerable households from the “silent crisis” of untreated chronic diseases.

## Data Availability

The data that support the findings of this study are available from the Somali National Bureau of Statistics (SNBS) microdata catalog at https://microdata.nbs.gov.so/index.php/catalog/50. Access will be granted upon reasonable request for legitimate research purposes.

## Appendix: Heckman probit sensitivity analysis

**Table:**

Variable	Coefficient	SE	*z*	*p*
Outcome: regular treatment				
Internet access	0.086	0.071	1.20	0.228
Secondary education or higher	0.195**	0.067	2.90	0.004
Climate shock	−0.091**	0.045	−2.05	0.040
Urban residence	0.168**	0.079	2.13	0.033
Selection: chronic disease				
Secondary education or higher	−0.106***	0.028	−3.77	< 0.001
Urban residence	0.154***	0.021	7.48	< 0.001
Age	0.027***	0.000	71.06	< 0.001
Model diagnostics				
Rho (ρ)	−0.318	0.357		
Prob > χ^2^	0.441			

Wealth score and Clean Fuel were included in the model estimations but omitted from this summary table for brevity. *p* < 0.05, *p* < 0.01, ****p* < 0.001.
